# Will things feel better in the morning? A time-of-day analysis of mental health and wellbeing from nearly 1 million observations

**DOI:** 10.1136/bmjment-2024-301418

**Published:** 2025-01-15

**Authors:** Feifei Bu, Jessica K Bone, Daisy Fancourt

**Affiliations:** 1 Research Department of Behavioural Science and Health, Institute of Epidemiology and Health Care, University College London, London, UK

**Keywords:** Anxiety disorders, PSYCHIATRY, Depression

## Abstract

**Background:**

Mood is known to change over seasons of the year, days of the week, and even over the course of the day (diurnally). But although broader mental health and well-being also vary over months and weeks, it is unclear whether there are diurnal changes in how people experience and report their mental health.

**Objective:**

To assess time-of-day association with depression, anxiety, well-being and loneliness.

**Methods:**

The study analysed data from 49 218 adults drawn from the University College London COVID-19 Social Study, which gathered detailed repeated measurements from the same participants across time over a 2-year period (March 2020–March 2022, 18.5 observation per person). Data were analysed using linear mixed-effects models.

**Findings:**

There is a clear time-of-day pattern in self-reported mental health and well-being, with people generally waking up feeling best and feeling worst around midnight. There is also an association with day of the week and season, with particularly strong evidence for better mental health and well-being in the summer. Time-of-day patterns are moderated by day, with more variation in mental health and individual well-being during weekends compared with weekdays. Loneliness is relatively more stable.

**Conclusions:**

Generally, things do seem better in the morning. Hedonic and eudemonic well-being have the most variation, and social well-being is most stable.

**Clinical implications:**

Our findings indicate the importance of considering time, day and season in research design, analyses, intervention delivery, and the planning and provision of public health services.

**WHAT IS ALREADY KNOWN ON THIS TOPIC**

- Mental health and well-being change over the life course, months and weeks. However, there are relatively few studies on diurnal changes.

**WHAT THIS STUDY ADDS**

- This study is unique in establishing associations of time-of-day with a range of mental health and well-being measures and assessing potential moderation effects by day of the week, season and year.

**HOW THIS STUDY MIGHT AFFECT RESEARCH, PRACTICE OR POLICY**

- Our findings indicate the importance of considering time, day and season in mental health and well-being research. These factors should also be considered for the design and delivery of interventions, as well as the planning and provision of public health services.

## Introduction

 Mental health and well-being (MHW) is dynamic in nature. A large and growing body of research has shown that MHW can change over both short and extended periods. For example, MHW is known to change over the life course,^1^,^3^ and recent studies examined how MHW fluctuated weekly or monthly during the COVID-19 pandemic.^4 5^ However, there are relatively few studies examining how MHW change over the course of the day (diurnally).

A theoretical rationale for diurnal changes in MHW comes from multiple domains. First, MHW is influenced by physiological processes, including neurotransmitters (eg, dopamine, serotonin), hormones (eg, cortisol) and inflammatory markers.^6 7^ Some of these physiological processes are known to change across the day. For example, cortisol typically peaks shortly after waking and decreases throughout the day, with the lowest levels between 8:00pm and 4:00am.^8 9^ Serotonin 5-HT_1A_ receptor and serotonin transporter 5-HTT have also been shown to vary across the day.^10^ Second, MHW is subject to the influences of geographical context and environmental factors (eg, sunlight, temperature, noise, air pollution),^11 12^ some of which may vary with systematic patterns across the day. Third, people tend to follow a relatively established sequence of daily activities (sleeping, waking, eating, working, commuting, exercising, socialising), which may to some extent shape the diurnal trajectory of MHW.^13^

Yet, empirical evidence on diurnal changes in MHW is lacking. Much of the existing evidence is on mood, a psychological concept that is closely related to MHW but focused on shorter-term affective states. But even the evidence on diurnal variation in mood is mixed and inconclusive, showing different patterns across the day.^13 14^ Inconsistent findings might be explained by the inclusion of small and biased samples (eg, university students) in studies. With the development of digital technology and data science, large-scale analyses have become increasingly feasible. An analysis of social media data showed that people tended to experience more positive affect at waking, which decreased throughout the day, but then started to increase again in the early evening.^15^ Negative affect was lowest in the morning and increased with time, peaking around midnight.^15^ These findings were supported by another study – this time on specific emotions – using social media data, which found that sadness and anger were lowest in the morning but increased throughout the day and peaked after midnight.^16^

Longer term, there is evidence of weekly and seasonal changes in mood. For example, at weekends, people typically report higher positive affect and circadian mood patterns are different compared with weekdays.^15 16^ There are seasonal patterns in signals of anger, anxiety, sadness and fatigue on social media, with anxiety peaking in spring and autumn and sadness peaking in winter.^17^ Alongside this, research has explored seasonal MHW variation. It has repeatedly been proposed that mental health is worst in winter and best in summer,^18^ with the extreme case being seasonal affective disorder.^19^ Although a systematic review concluded that findings are inconsistent,^20^ more recent large studies using electronic health records have supported this theory for various psychiatric disorders across different countries.^18 21^ This is also reflected in online behaviour, with Google searches for mental health information most common in winter and least common in summer.^18 22^ However, it remains unclear whether diurnal patterns of mood are reflected in diurnal changes in self-reported MHW, and whether any diurnal changes in MHW might be related to, or influenced by, longer-term patterns of MHW.

Therefore, this study aimed to examine whether time of day was associated with MHW, including mental health (depressive symptoms and anxiety symptoms), hedonic well-being comprising experienced well-being (happiness) and evaluative well-being (life satisfaction), eudemonic well-being (sense of life being worthwhile) and social well-being (loneliness). Further, it examined whether these associations vary across different days, seasons and years. These questions have important implications for study design and analysis, as well as the delivery of mental health interventions and public health services.

## Methods

### Data

This study analysed data from the University College London (UCL) COVID-19 Social Study (CSS), a large panel study of over 73 000 adults (aged 18+ years) in the UK. The study commenced on 21 March 2020 with weekly then monthly follow-ups until November 2021, and additional follow-ups in November 2021, and January and March 2022. Participants were recruited through existing mailing lists (including large databases of adults who had previously consented to be involved in health research), print and digital media coverage, and social media. This was supplemented by targeted recruitments focusing on underrepresented groups via advertising/recruitment companies, and third-sector organisations. Full details of sample recruitment are published elsewhere.^23^ Participants provided an email address on first enrolment, which was used for receiving invites for follow-up surveys. During the weekly follow-up stage, survey invites were sent at the same time of day 1 week after the last survey completion. At the start of the monthly follow-up stage, participants were randomly allocated into four groups to receive their first monthly invite in different weeks on Monday morning (8:30am–12:30pm). Monthly follow-up survey invites were sent at the same time 4 weeks after last survey completion. At the additional follow-up stages, survey invites were sent on 22 November 2021 (Monday, 11:00am–12:00pm), 4 January 2021 (Tuesday, 9:30am–10:30am) and 21 March 2022 (Monday, 8:00am–9:30am). For each survey, a daily reminder was sent out automatically up to two times for those who did not respond to the initial invite. If participants did not complete the survey following the two reminders they would stop receiving future invites.

This analysis focused on participants living in England (n=59 810). After excluding participants with missing demographic, socioeconomic and health data at baseline, we had an analytical sample of 49 218 unique participants. The study was particularly valuable for exploring time-of-day association with MHW as participants completed the same core battery of questionnaires multiple times across 2 years, during which there was natural variation in the time of day that people responded, prompts across different days of the week, and variations in seasons. On average, participants in the sample provided 908 769 total observations across 2 years (range, 825 410 to 929 813 depending on the outcome measure), with substantial variation in when they responded (online supplemental table S1).

### Measurements

#### Outcomes

Depressive symptoms were measured using the Patient Health Questionnaire (PHQ-9), a standardised instrument for screening for depression in primary care. The current study measured symptoms ‘over the last week’ instead of ‘over the last 2 weeks’ (as in the original PHQ-9), as data in the study were initially collected weekly. The questionnaire includes nine items with four-point responses ranging from ‘not at all’ to ‘nearly every day’. The raw sum score ranged from 0 to 27, with higher values indicating more depressive symptoms.

Anxiety symptoms were measured using the Generalised Anxiety Disorder assessment (GAD-7), a validated tool used to screen for generalised anxiety disorder in clinical practice and research. As with the PHQ-9, this questionnaire also measured symptoms ‘over the last week’. The GAD-7 comprises seven items with four-point responses ranging from ‘not at all’ to ‘nearly every day’. The raw sum score ranged from 0 to 21, with higher values indicating more anxiety symptoms.

Well-being was measured using items adapted from the UK Office for National Statistics personal well-being questions. Happiness (experienced well-being) was measured by a single question on a scale of 0 to 10: ‘In the past week, how happy did you feel?’. Life satisfaction (evaluative well-being) was measured by a single question on a scale of 0 to 10: ‘Overall, in the past week, how satisfied have you been with your life?’. Sense of life being worthwhile (eudemonic well-being) was measured by a single question on a scale of 0 to 10: ‘In the past week, to what extent have you felt the things you are doing in your life are worthwhile?’. Higher scores indicate greater well-being.

Loneliness (social well-being) was measured using the three-item UCLA Loneliness Scale (UCLA-3). Responses to each question were scored on a three-point Likert scale (hardly ever/never, some of the time, often). The raw sum score ranged from 3 to 9, with a higher score indicating higher loneliness.

All outcome measures were standardised to have a mean of 0 and a SD of 1.

#### Exposures

Exposure variables included time of day (continuous from 6am to midnight), day of the week (categorical, Monday to Sunday), astronomical season (Spring, Summer, Autumn, Winter based on equinoxes and solstices, see online supplemental table S2) and survey year (2020, 2021, 2022), all of which were generated from timestamps when participants completed each survey. Time was restricted to typical waking hours between 6:00am and midnight as only a small number of people responded outside these hours (see online supplemental figure S1).

#### Covariates

Covariates were all measured at baseline, including age groups (18–29, 30–45, 46–59, 60+ years), gender (woman, man), ethnicity (non-White, White), education (low: General Certificate of Secondary Education (GCSE) or below, medium: A levels or equivalent, high: degree or above), employment status (employed, other), residential area (rural, urban), diagnosed physical health condition (yes, no) and diagnosed mental health condition (yes, no).

#### Analysis

Data were analysed using linear mixed-effects models, testing the effect of time of day (time), day of the week, season and year on each of the six outcomes in separate models. Quadratic and cubic terms of time were included to allow for nonlinear trajectories. Models were adjusted for all time-invariant covariates (see Statistical modelling in the online supplemental materials for technical explanations). Further, we tested whether the association of time with each outcome differed according to day of the week, astronomical season and survey year. We tested this by adding an interaction term between time and each potential moderator.

To account for the non-random nature of the sample, the sample was weighted using entropy balancing weights^24^ for the English population proportions of gender, age, ethnicity and education obtained from the Office for National Statistics. All analyses were conducted using Stata V18.

## Results

In the raw data of 49 218 participants, 76.4% were women and there was an overrepresentation of people with a high level of education (degree or above, 68.1%) and an under-representation of people from ethnic minority backgrounds (5.9%; table 1). After weighting, the sample reflected population proportions, with 50.8% women, 34.2% of participants with high education and 14.6% of ethnic minority. The between and within variation of time and outcome measures are reported in online supplemental table S1. About 84.4% of total variance in time was within individuals. There were higher within-individual variations in well-being measures (happiness 32.3%, life satisfaction 34.1%, worthwhile 34.2%) compared with depressive (23.9%) and anxiety symptoms (24.9%). Loneliness had the lowest within-individual variance (19.6%).

**Table 1:** Sample characteristics (n=49 218)

Characteristic	Before weighting (%)	After weighting (%)
Age: 18–29 years	8.9	19.6
Age: 30–45 years	29.6	26.4
Age: 46–59 years	32.7	24.0
Age: 60+ years	28.8	30.0
Gender: women	76.4	50.8
Gender: men	23.6	49.2
Ethnicity: minority	5.9	14.6
Ethnicity: white	94.1	85.4
Education: low	14.1	32.7
Education: medium	17.7	33.1
Education: high	68.1	34.2
Employment: employed	65.8	58.3
Employment: other	34.2	41.7
Area of living: urban	79.3	80.8
Area of living: rural	20.7	19.2
Living status: alone	18.2	17.3
Living status: not alone	81.8	82.7
Physical health condition: yes	38.9	41.0
Poor physical health: no	61.1	59.0
Poor mental health: yes	18.8	20.2
Poor physical health: no	81.2	79.8

### Associations of time with MHW

**Figure 1:**
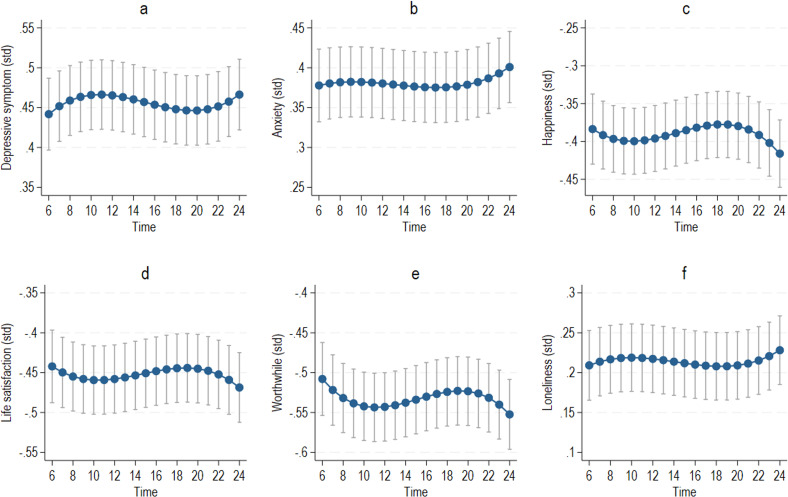
Predicted changes over time from linear mixed-effects model for each outcome (covariates fixed at reference values): (**a**) depressive symptoms, (**b**) anxiety symptoms, (**c**) happiness, (**d**) life satisfaction, (**e**) worthwhile and (**f**) loneliness. std, standardised.

Figure 1 shows the predicted trajectories over time for each outcome and their 95% confidence intervals (full results are in online supplemental table S3). After controlling for covariates, there was evidence for a nonlinear relationship between time and MHW. Although the differences across time were small, outcomes were consistently best early in the day (lowest depressive and anxiety symptoms and loneliness and highest happiness, life satisfaction and worthwhile ratings) and worst late at night. Worthwhile ratings had the most variation, peaking early in the morning, then reducing until midday, followed by a second lower peak in the evening, and a sharp decrease to its lowest at midnight.

**Figure 2:**
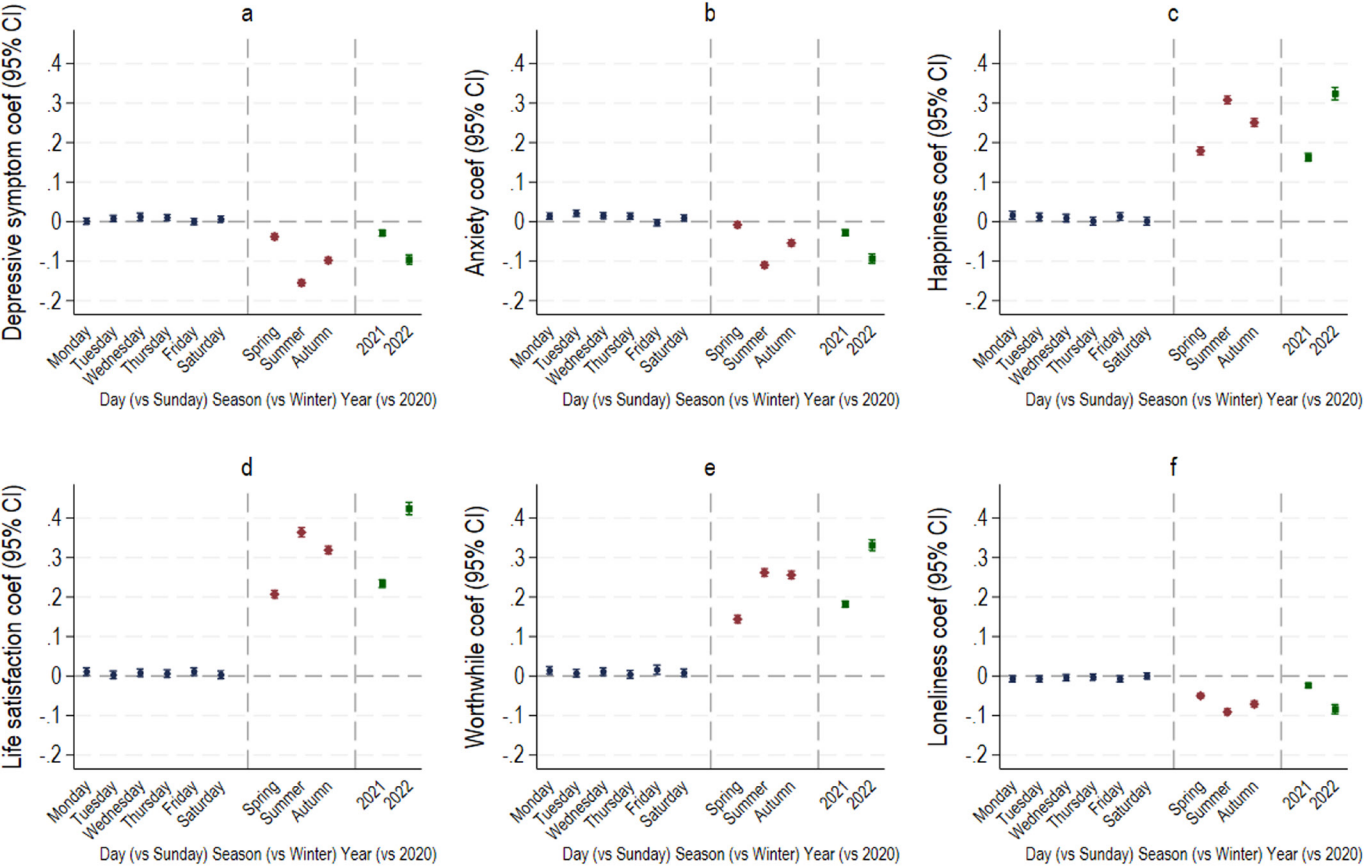
Coefficients and 95% confidence intervals from linear mixed-effects model showing average differences by day, season and year for each outcome: (**a**) depressive symptoms, (**b**) anxiety symptoms, (**c**) happiness, (**d**) life satisfaction, (**e**) worthwhile and (**f**) loneliness. Reference categories were Sunday (day), winter (season) and 2020 (year).

There was some inconsistent evidence that day of the week was associated with MHW (figure 2). For example, compared with Sunday, depressive symptoms were higher on Wednesdays and Thursdays. Anxiety symptoms were higher on all days versus Sunday except Fridays. Happiness, life satisfaction and worthwhile ratings were all higher on Mondays and Fridays than Sundays, and happiness was also higher on Tuesdays. However, there was no evidence that loneliness differed across days of the week.

There was much more consistent evidence for a seasonal effect. Compared with winter, individuals tended to have lower levels of depressive and anxiety symptoms and loneliness, and higher levels of happiness, life satisfaction and worthwhile in other seasons. MHW was best in the summer across all outcomes. Moreover, survey year was associated with all outcomes, indicating steady improvements in MHW since 2020, the first year of the COVID-19 pandemic.

### Moderation by day, season and year

**Figure 3:**
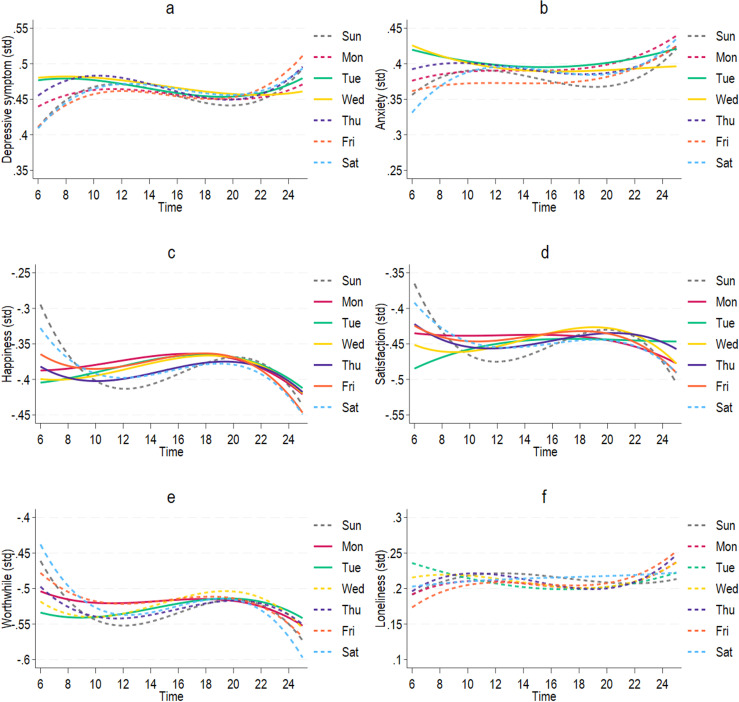
Predicted changes over time from linear mixed-effects model with moderation by day of the week for each outcome (covariates fixed at reference values): (**a**) depressive symptoms, (**b**) anxiety symptoms, (**c**) happiness, (**d**) life satisfaction, (**e**) worthwhile and (**f**) loneliness. Solid lines representing significantly different patterns to Sunday (p<0.05). std, standardised.

We then tested whether the association of time with each outcome differed across day of the week, astronomical season and survey year. Only the interactions between time and day of the week were statistically significant and so were included in the final model (online supplemental table S4). Figure 3 shows the predicted patterns over time separately by day of the week for each outcome, setting other covariates at their reference values.

For depressive and anxiety symptoms, Tuesday and Wednesday showed different patterns of change compared with Sunday. On these 2 days, depressive and anxiety symptoms were higher in the morning, and decreased slightly throughout the day, before starting to increase into the night. However, on other days, depressive and anxiety symptoms were at the lowest level in the morning, fluctuated throughout the day, and then peaked at midnight. The difference between early morning and midnight could be as high as 10% of the SD for depressive and anxiety symptoms. Relative to other days, there were fewer changes across time on Tuesday and Wednesday.

There were substantial differences between early morning and midnight in well-being measures, which were as high as 15% of the SD. For happiness and life satisfaction, the weekdays showed fewer variations across time compared with weekends. During weekends, both happiness and life satisfaction were at their highest in the morning, decreased sharply until midday, increased steadily into the evening, and then started to decrease again, with the lowest point at midnight. Worthwhile ratings showed similar patterns on weekends. But, in contrast to other well-being outcomes, worthwhile ratings on Wednesday to Friday followed similar patterns to the weekend. Only Monday and Tuesday were significantly different to Sunday, showing less variation across time.

Loneliness was relatively stable across time, and there were no differences in time-of-day patterns across different days of the week.

## Discussion

This study found that self-reported MHW varied across time of the day, with people generally waking up with better MHW and feeling worst around midnight. Both day of the week and season were also associated with MHW, with particularly strong seasonal effects favouring the summer. Moderation effects were only observed for day of the week. Overall, there appeared to be more time-of-day variation during weekends compared with weekdays. Loneliness was the exception to this, as it remained more stable over time of day and day of the week.

Our findings on time of day are largely similar to previous findings on mood,^15 16^ as people tend to have better MHW in the morning and worst around midnight, particularly during weekends. This might be explained by circadian rhythms in physiological processes. For example, cortisol peaks shortly after waking and reaches its lowest levels around bedtime.^9^ However, it is important to acknowledge the differences between weekends and weekdays. Given there is little evidence that physiological processes differ across different days of the week, differences might be related to other factors that drive MHW changes over the course of the day. This could include contextual factors and sequence of daily activities, which are likely to be different between weekends and weekdays.

We found much clearer evidence for seasonal changes in MHW, in line with previous evidence for winter highs and summer lows in symptoms, medication prescriptions, admissions and social media searches for various mental health problems.^18 21 22^ It is important to note that all our MHW measures showed these seasonal changes, indicating that both positive and negative, hedonic and eudemonic, individual and social aspects of well-being change in tandem throughout the year. We also provide the first data indicating that diurnal variations in MHW do not differ by season. This is somewhat surprising given that one of the main explanations for seasonal changes in MHW is due to meteorological cycles, including number of daylight hours, which we might expect to influence diurnal changes in MHW.^18^ Other drivers of the seasonal variation in MHW could include weather (temperature, precipitation, humidity) as well as various sociocultural cycles, including cultural holidays, norms and employment patterns.^18^ How these factors relate to diurnal, short-term and long-term changes in MHW requires further investigation, but it appears they are not key drivers of diurnal variations.

Overall, among all MHW outcomes, there was a general curvilinear pattern across the day. However, there were subtle differences across psychological domains when testing how time-of-day patterns differed by day of the week. Measures of mental health were worst mid-week, with morning relief from depressive and anxiety symptoms not found on Tuesdays and Wednesdays. When compared with evidence that suicide risk peaks on Monday,^25^ it is surprising that we did not also find elevated mental health symptoms on Monday. However, in line with our findings, suicide risk remains elevated across weekdays and is lowest Friday to Sunday.^25^ Previous research has linked changes in MHW across the week to work stress, with employment leading to reductions in MHW during weekdays compared with weekends.^26^ Supporting the hypothesis that work may be driving these results, we found less time-of-day variation in eudemonic well-being (feeling life was worthwhile) at the start of the working week, as it did not have the morning peak found at the weekend and later in the week. Hedonic well-being measures (happiness and life satisfaction) showed clearer differentiation between the weekend and weekdays, with scores peaking first thing in the morning only at the weekend. This could be in anticipation of a day of leisure, as the weekend peak in MHW is associated with doing more social leisure activities at the weekend^27^ and the difference in time spent with friends or family between weekends and weekdays.^26^

In comparison with measures of individual mental health and well-being, loneliness (which focuses on social aspects of well-being) showed the smallest variation across time of day, no substantial difference across day of the week, and the least variation over seasons. This is not surprising given loneliness has been found to be stable over extended periods or even life course.^28 29^ Compared with other measures, loneliness has trait-like features similar to aspects of personality.^28^ Individual well-being measures showed the greatest overall variability. This could be a true effect, whereby well-being varies more over time than the more moderate fluctuations in depressive and anxiety symptoms, which are driven by underlying disorders. However, it could also be an artefact of the instruments used. Symptoms were measured with seven- and nine-item questionnaires, developed and validated to provide consistent measures over time, with responses measured on four-point Likert scales. Individual well-being, in contrast, was measured with single questions and continuous 10-point rating scales, which may encourage more variability in responses. Additionally, as these well-being questions ask for a global assessment of the past week (‘overall, in the past week…’), there may be a mood (or indeed mental health) effect on responses, such that people’s view of the past week may be positively or negatively biased by the current mood they are experiencing. Future research should therefore compare variations in mental health and well-being using similar in-depth validated measures that include more objective assessments of the number of days particular symptoms have been experienced rather than merely a single global judgement on experiences.

These findings have important implications in three key areas. First, within research design, it is important to account for time, day and season when examining changes in MHW. In observational research, this information is usually available from online data collection or computer-assisted personal interviews (CAPI), but is not usually released, as it is often considered as sensitive or non-essential. Nonetheless, these findings suggest the importance of incorporating such data into study design and ethics plans. Equally, for experimental studies, it has long been established that the timing of biological measures (eg, cortisol) and their follow-ups must be carefully planned across the day. While our findings do not point to a specific time being optimal for capturing MHW data, they do show that measures vary across time of day, day of the week and season. Consideration of this within study design will be particularly important for small sample sizes, non-randomised quasi-experimental studies, or those offering interventions at differing times of year.

In terms of interventions, understanding diurnal variation is important for the delivery of services. Data on calls to Samaritans helplines show similar patterns across time of day for ‘regular callers’ and similar increases across the afternoon into the evening for ‘typical callers’, the two largest categories of callers (although other smaller categories of callers have alternative patterns).^30^ Our findings demonstrating the likely fluctuations in people’s MHW patterns could inform training of staff involved in 24-hour services to support their conversations with patients/callers. The findings presented here also have implications for clinical assessments, suggesting that mental health screening could result in different outcomes depending on when that screening occurs. Whether people are more receptive to interventions at different points in their diurnal mental health cycle remains to be explored. Finally, in relation to public health, our findings indicate that people’s MHW tends to be lowest around midnight, mid-week and in winter. This should be considered when planning service and resource provision, such as mental health crisis services. Longer-term patterns could also be relevant to individual treatment plans, anticipating greater clinical input at these times.

This study has several strengths. We were able to test patterns over nearly a million observations of MHW, exploring the collective effects of time, day of the week, season and year. We determined these time variables from accurate survey timestamps, using equinoxes and solstices to determine astronomical season. The UCL COVID-19 Social Study did not use a random sample, but it does have a large sample size with wide heterogeneity, including good stratification across all major sociodemographic groups. In addition, analyses were weighted using population estimates of core demographics, resulting in good alignment with national population statistics. However, we cannot rule out the possibility of potential biases due to omitting other demographic factors that could be associated with survey participation in the weighting process. Although we adjusted for several time-invariant covariates, residual confounding (particularly of time-varying confounders) is possible. In particular, we do not know why people chose to respond at different times of day or days of the week, which arguably might be affected by their MHW. In other words, we cannot rule out the possibility of reverse causality, and these associations should not be overinterpreted as causal relationships. Ecological momentary assessment studies are encouraged to confirm the validity of our findings at multiple random times within the same day. Such studies should also assess directionality, exploring whether time of day leads to different states of MHW (eg, early morning being a time that people generally feel better) or whether people with better MHW have different sleep cycles (eg, waking and going to bed early). There are also other moderators that may influence findings. For example, our sample was limited to England, meaning we could not test the effects of latitude and weather patterns, which are thought to modify seasonal patterns. Our findings therefore cannot be generalised to other geographical locations.

Overall, we found that self-reported MHW differed according to time of day, day of the week and season of the year, with time-of-day associations also differing across days of the week. Generally, things do indeed seem better in the morning. People generally reported the worst MHW late in the day and in winter, and there was more variation at the weekends. Looking across different aspects of MHW, hedonic and eudemonic well-being had the most variation, and social well-being was most stable. Our findings indicate the importance of considering time, day and season in both the design and analysis of MHW research. These factors should also be considered for the design and delivery of interventions, as well as the planning and provision of public health services.

## supplementary material

10.1136/bmjment-2024-301418 online supplemental file 1

## Data Availability

Data are available in a public, open access repository.

## References

[1] Bell A (2014). Life-course and cohort trajectories of mental health in the UK, 1991-2008--a multilevel age-period-cohort analysis. Soc Sci Med.

[2] Galambos NL, Krahn HJ, Johnson MD (2020). The U Shape of Happiness Across the Life Course: Expanding the Discussion. Perspect Psychol Sci.

[3] George LK (2013). Handbooks of sociology and social research.

[4] Fancourt D, Steptoe A, Bu F (2021). Trajectories of anxiety and depressive symptoms during enforced isolation due to COVID-19 in England: a longitudinal observational study. Lancet Psychiatry.

[5] Bu F, Steptoe A, Fancourt D (2023). Depressive and anxiety symptoms in adults during the COVID-19 pandemic in England: A panel data analysis over 2 years. PLoS Med.

[6] Lin SH, Lee LT, Yang YK (2014). Serotonin and mental disorders: a concise review on molecular neuroimaging evidence. Clin Psychopharmacol Neurosci.

[7] de Vries LP, van de Weijer MP, Bartels M (2022). The human physiology of well-being: A systematic review on the association between neurotransmitters, hormones, inflammatory markers, the microbiome and well-being. Neurosci Biobehav Rev.

[8] Weitzman ED, Fukushima D, Nogeire C (1971). Twenty-four hour pattern of the episodic secretion of cortisol in normal subjects. J Clin Endocrinol Metab.

[9] Dmitrieva NO, Almeida DM, Dmitrieva J (2013). A day-centered approach to modeling cortisol: diurnal cortisol profiles and their associations among U.S. adults. Psychoneuroendocrinology.

[10] Matheson GJ, Schain M, Almeida R (2015). Diurnal and seasonal variation of the brain serotonin system in healthy male subjects. Neuroimage.

[11] Mullins JT, White C (2019). Temperature and mental health: Evidence from the spectrum of mental health outcomes. J Health Econ.

[12] Rautio N, Filatova S, Lehtiniemi H (2018). Living environment and its relationship to depressive mood: A systematic review. Int J Soc Psychiatry.

[13] Stone AA, Schwartz JE, Schkade D (2006). A population approach to the study of emotion: diurnal rhythms of a working day examined with the Day Reconstruction Method. Emotion.

[14] Scheer FAJL, Chellappa SL (2024). Endogenous circadian rhythms in mood and well-being. Sleep Health.

[15] Golder SA, Macy MW (2011). Diurnal and Seasonal Mood Vary with Work, Sleep, and Daylength Across Diverse Cultures. Science.

[16] Dzogang F, Lightman S, Cristianini N (2017). Circadian mood variations in Twitter content. Brain Neurosci Adv.

[17] Dzogang F, Goulding J, Lightman S (2017). Seasonal variation in collective mood via Twitter content and medical purchases.

[18] Hohm I, Wormley AS, Schaller M (2024). *Homo temporus*: Seasonal Cycles as a Fundamental Source of Variation in Human Psychology. Perspect Psychol Sci.

[19] Magnusson A, Boivin D (2003). Seasonal affective disorder: an overview. Chronobiol Int.

[20] Øverland S, Woicik W, Sikora L (2019). Seasonality and symptoms of depression: A systematic review of the literature. Epidemiol Psychiatr Sci.

[21] Törmälehto S, Svirskis T, Partonen T (2022). Seasonal Effects on Hospitalizations Due to Mood and Psychotic Disorders: A Nationwide 31-Year Register Study. Clin Epidemiol.

[22] Ayers JW, Althouse BM, Allem J-P (2013). Seasonality in seeking mental health information on Google. Am J Prev Med.

[23] Fancourt D, Bu F, Paul E (2022). UCL COVID-19 Social Study, 2020–2022.

[24] Hainmueller J, Xu Y (2013). Ebalance: A Stata Package for Entropy Balancing. J Stat Softw.

[25] Cavanagh B, Ibrahim S, Roscoe A (2016). The timing of general population and patient suicide in England, 1997-2012. J Affect Disord.

[26] Helliwell JF, Wang S (2014). Weekends and Subjective Well-Being. Soc Indic Res.

[27] Fritz C, Sonnentag S (2005). Recovery, health, and job performance: effects of weekend experiences. J Occup Health Psychol.

[28] Mund M, Freuding MM, Möbius K (2020). The Stability and Change of Loneliness Across the Life Span: A Meta-Analysis of Longitudinal Studies. Pers Soc Psychol Rev.

[29] Bu F, Steptoe A, Fancourt D (2020). Loneliness during a strict lockdown: Trajectories and predictors during the COVID-19 pandemic in 38,217 United Kingdom adults. Soc Sci Med.

[30] Turkington R, Mulvenna M, Bond R (2020). Behavior of Callers to a Crisis Helpline Before and During the COVID-19 Pandemic: Quantitative Data Analysis. JMIR Ment Health.

