# Supplementary material for: Will things feel better in the morning? A time-of-day analysis of mental health and wellbeing from nearly 1 million observations
Source: BMJ Ment Health. 2025 Jan 15;28(1):e301418. doi: 10.1136/bmjment-2024-301418 (PMC11795389; doi:10.1136/bmjment-2024-301418)

# **Supplementary Materials**

**Statistical modelling**

Given that people never gave more than one survey response on a single day, we modelled both within- and between-individual variation, with the outcomes varying at both levels. We parameterised the models as:

$y_{it}=\pi_{0i}+\pi_{1i}T_{it}+ \pi_{2}T_{it}^{2}+ \pi_{3}T_{it}^{3} + \pi_{4}X_{it} {+ \pi_{5}Z_{i} + \varepsilon}_{it}$ (Eq. 1a)

$\pi_{0i}=\beta_{00}+\zeta_{0i}$ (Eq. 1b)

$\pi_{1i}=\beta_{10}+\zeta_{1i}$ (Eq. 1c)

$\varepsilon_{it}\sim N(0, \sigma_{\varepsilon}^{2})$ (Eq. 1d)

$\left[ \begin{aligned} \zeta_{0i} \\ \zeta_{1i} \end{aligned} \right]\sim\left( \left[ \begin{aligned} 0 \\ 0 \end{aligned} \right],\left[ \begin{matrix} \sigma_{0}^{2} & \sigma_{01} \\ \sigma_{10} & \sigma_{1}^{2} \end{matrix} \right] \right)$ (Eq. 1e)

Where $y_{it}$ is the outcome variable for participant *i* at time *t*. $\pi_{0i}$ is the individual-specific intercept, with a variance of $\sigma_{0}^{2}$ (Eq. 1e). $T_{it}$ is the time variable. Polynomial terms are included to allow for non-linear relationships. For model parsimony, only the linear coefficient $\pi_{1i}$ is set as random across individuals, with a variance of $\sigma_{1}^{2}$. $X_{it}$ represents a vector of time-varying covariates that might be associated with outcome, such as day of the week, season, and year. $Z_{i}$ represents a vector of time-invariant control variables (e.g. age, gender). $\varepsilon_{it}$ is the level 1 residual, with a mean of 0 and variance of $\sigma_{\varepsilon}^{2}$ (Eq. 1c).

**Table S1.** Descriptive statistics for the time of the day variable and outcome measures.

|  |  | SD | ρ | N (observations)/  n (individuals) |
| --- | --- | --- | --- | --- |
| Hour | Between | 1.823 | 0.156 | 930,758 |
|  | Within | 4.241 |  | 49,218 |
| Day of week | Between | 0.435 | 0.060 | 930,758 |
|  | Within | 1.727 |  | 49,218 |
| Season | Between | 0.000 | 0.000 | 930,758 |
|  | Within | 0.855 |  | 49,218 |
| Year | Between | 0.156 | 0.070 | 930,758 |
|  | Within | 0.567 |  | 49,218 |
| Depressive symptoms | Between | 0.859 | 0.761 | 924,367 |
|  | Within | 0.481 |  | 49,218 |
| Anxiety symptoms | Between | 0.856 | 0.751 | 922,141 |
|  | Within | 0.493 |  | 49,218 |
| Happiness | Between | 0.815 | 0.677 | 825,410 |
|  | Within | 0.563 |  | 49,218 |
| Life satisfaction | Between | 0.802 | 0.659 | 929,813 |
|  | Within | 0.577 |  | 49,218 |
| Worthwhile | Between | 0.802 | 0.658 | 929,810 |
|  | Within | 0.578 |  | 49,218 |
| Loneliness | Between | 0.891 | 0.804 | 921,074 |
|  | Within | 0.440 |  | 49,218 |

**Table S2** Solstices and Equinoxes dates in the UK 2020-2022

| Year | Event | Date |
| --- | --- | --- |
| 2020 | Spring Equinox | 20 Mar 2020 |
|  | Summer Solstice | 20 Jun 2020 |
|  | Autumn Equinox | 22 Sep 2020 |
|  | Winter Solstice | 21 Dec 2020 |
| 2021 | Spring Equinox | 20 Mar 2021 |
|  | Summer Solstice | 21 Jun 2021 |
|  | Autumn Equinox | 22 Sep 2021 |
|  | Winter Solstice | 21 Dec 2021 |
| 2022 | Spring Equinox | 20 Mar 2022 |
|  | Summer Solstice | 21 Jun 2022 |
|  | Autumn Equinox | 23 Sep 2022 |
|  | Winter Solstice | 21 Dec 2022 |

Source: https://greenwichmeantime.com/

**Table S3a.** Results from linear mixed-effects models on depressive symptoms (PHQ), anxiety symptoms (GAD), and happiness.

|  | Depressive symptoms | | | Anxiety symptoms | | | Happiness | | |
| --- | --- | --- | --- | --- | --- | --- | --- | --- | --- |
| Fixed estimates | Coef. | SE | p | Coef. | SE | p | Coef. | SE | p |
| Time | 0.038 | (0.007) | <0.001 | 0.014 | (0.007) | 0.051 | -0.037 | (0.009) | <0.001 |
| Time^2^ | -0.003 | (0.000) | <0.001 | -0.001 | (0.000) | 0.016 | 0.003 | (0.001) | <0.001 |
| Time^3^ | 0.000 | (0.000) | <0.001 | 0.000 | (0.000) | 0.004 | -0.000 | (0.000) | <0.001 |
| Mon (vs. Sun) | 0.001 | (0.004) | 0.858 | 0.014 | (0.004) | <0.001 | 0.016 | (0.005) | 0.001 |
| Tue (vs. Sun) | 0.008 | (0.004) | 0.067 | 0.021 | (0.004) | <0.001 | 0.012 | (0.005) | 0.025 |
| Wed (vs. Sun) | 0.012 | (0.005) | 0.010 | 0.015 | (0.004) | 0.001 | 0.009 | (0.005) | 0.107 |
| Thu (vs. Sun) | 0.010 | (0.004) | 0.019 | 0.014 | (0.004) | 0.001 | 0.001 | (0.005) | 0.911 |
| Fri (vs. Sun) | 0.000 | (0.004) | 0.982 | -0.003 | (0.004) | 0.484 | 0.013 | (0.005) | 0.017 |
| Sat (vs. Sun) | 0.006 | (0.004) | 0.142 | 0.009 | (0.004) | 0.027 | 0.001 | (0.005) | 0.767 |
| Spring (vs. Winter) | -0.038 | (0.004) | <0.001 | -0.008 | (0.004) | 0.034 | 0.179 | (0.005) | <0.001 |
| Summer (vs. Winter) | -0.155 | (0.004) | <0.001 | -0.110 | (0.004) | <0.001 | 0.308 | (0.005) | <0.001 |
| Autumn (vs. Winter) | -0.098 | (0.004) | <0.001 | -0.054 | (0.004) | <0.001 | 0.251 | (0.005) | <0.001 |
| Year 2021 (vs. 2020) | -0.029 | (0.004) | <0.001 | -0.028 | (0.004) | <0.001 | 0.163 | (0.005) | <0.001 |
| Year 2022 (vs. 2020) | -0.096 | (0.006) | <0.001 | -0.094 | (0.006) | <0.001 | 0.324 | (0.008) | <0.001 |
| Constant | 0.301 | (0.040) | <0.001 | 0.330 | (0.040) | <0.001 | -0.252 | (0.048) | <0.001 |
| Random estimates |  |  |  |  |  |  |  |  |  |
| $\sigma_{1}^{2}$ | 0.000 | (0.000) | <0.001 | 0.000 | (0.000) | <0.001 | 0.000 | (0.000) | <0.001 |
| $\sigma_{0}^{2}$ | 0.666 | (0.009) | <0.001 | 0.696 | (0.009) | <0.001 | 0.646 | (0.008) | <0.001 |
| $\sigma_{01}$ | -0.005 | (0.000) | <0.001 | -0.006 | (0.000) | <0.001 | -0.005 | (0.000) | <0.001 |
| $\sigma_{\varepsilon}^{2}$ | 0.218 | (0.002) | <0.001 | 0.220 | (0.002) | <0.001 | 0.292 | (0.003) | <0.001 |
| Number of individuals (n) | 49,218 |  |  | 49,218 |  |  | 49,218 |  |  |
| Number of observations (N) | 924,367 |  |  | 922,141 |  |  | 825,410 |  |  |

*Note:* All time-invariant covariates (age, gender, ethnicity, education, area of living, living status, physical health, mental health) were included in each model, but estimates are not reported to avoid Table 2 fallacy.

**Table S3b.** Results from linear mixed-effects models on life satisfaction, worthwhile, and loneliness.

|  | Life Satisfaction | | | Worthwhile | | | Loneliness | | |
| --- | --- | --- | --- | --- | --- | --- | --- | --- | --- |
| Fixed estimates | Coef. | SE | p | Coef. | SE | p | Coef. | SE | p |
| Time | -0.031 | (0.009) | 0.001 | -0.050 | (0.009) | <0.001 | 0.020 | (0.006) | 0.002 |
| Time^2^ | 0.002 | (0.001) | <0.001 | 0.004 | (0.001) | <0.001 | -0.002 | (0.000) | <0.001 |
| Time^3^ | -0.000 | (0.000) | <0.001 | -0.000 | (0.000) | <0.001 | 0.000 | (0.000) | <0.001 |
| Mon (vs. Sun) | 0.011 | (0.005) | 0.024 | 0.014 | (0.005) | 0.003 | -0.007 | (0.004) | 0.053 |
| Tue (vs. Sun) | 0.003 | (0.005) | 0.522 | 0.007 | (0.005) | 0.203 | -0.007 | (0.004) | 0.064 |
| Wed (vs. Sun) | 0.008 | (0.005) | 0.100 | 0.011 | (0.005) | 0.036 | -0.004 | (0.004) | 0.293 |
| Thu (vs. Sun) | 0.006 | (0.005) | 0.231 | 0.004 | (0.005) | 0.515 | -0.003 | (0.004) | 0.482 |
| Fri (vs. Sun) | 0.011 | (0.005) | 0.038 | 0.016 | (0.006) | 0.004 | -0.007 | (0.004) | 0.094 |
| Sat (vs. Sun) | 0.003 | (0.005) | 0.512 | 0.008 | (0.005) | 0.133 | 0.000 | (0.004) | 0.946 |
| Spring (vs. Winter) | 0.207 | (0.005) | <0.001 | 0.144 | (0.005) | <0.001 | -0.050 | (0.003) | <0.001 |
| Summer (vs. Winter) | 0.364 | (0.006) | <0.001 | 0.262 | (0.005) | <0.001 | -0.091 | (0.004) | <0.001 |
| Autumn (vs. Winter) | 0.319 | (0.005) | <0.001 | 0.256 | (0.005) | <0.001 | -0.071 | (0.004) | <0.001 |
| Year 2021 (vs. 2020) | 0.234 | (0.005) | <0.001 | 0.182 | (0.004) | <0.001 | -0.024 | (0.003) | <0.001 |
| Year 2022 (vs. 2020) | 0.424 | (0.008) | <0.001 | 0.331 | (0.007) | <0.001 | -0.084 | (0.006) | <0.001 |
| Constant | -0.330 | (0.047) | <0.001 | -0.318 | (0.049) | <0.001 | 0.137 | (0.037) | <0.001 |
| Random estimates |  |  |  |  |  |  |  |  |  |
| $\sigma_{1}^{2}$ | 0.000 | (0.000) | <0.001 | 0.000 | (0.000) | <0.001 | 0.000 | (0.000) | <0.001 |
| $\sigma_{0}^{2}$ | 0.656 | (0.008) | <0.001 | 0.662 | (0.008) | <0.001 | 0.701 | (0.006) | <0.001 |
| $\sigma_{01}$ | -0.007 | (0.000) | <0.001 | -0.007 | (0.000) | <0.001 | -0.004 | (0.000) | <0.001 |
| $\sigma_{\varepsilon}^{2}$ | 0.304 | (0.003) | <0.001 | 0.331 | (0.003) | <0.001 | 0.184 | (0.002) | <0.001 |
| Number of individuals (n) | 49,218 |  |  | 49,218 |  |  | 49,218 |  |  |
| Number of observations (N) | 929,813 |  |  | 929,810 |  |  | 921,074 |  |  |

*Note:* All time-invariant covariates (age, gender, ethnicity, education, area of living, living status, physical health, mental health) were included in each model, but estimates are not reported to avoid Table 2 fallacy.

**Table S4a.** Results from linear mixed-effects models testing whether associations between depressive symptoms (PHQ), anxiety symptoms (GAD), and happiness were moderated by day of the week.

|  | Depressive symptoms | | | Anxiety symptoms | | | Happiness | | |
| --- | --- | --- | --- | --- | --- | --- | --- | --- | --- |
| Fixed estimates | Coef. | SE | p | Coef. | SE | p | Coef. | SE | p |
| Time | 0.075 | (0.021) | <0.001 | 0.053 | (0.019) | 0.005 | -0.127 | (0.025) | <0.001 |
| Time^2^ | -0.005 | (0.001) | <0.001 | -0.004 | (0.001) | 0.002 | 0.008 | (0.002) | <0.001 |
| Time^3^ | 0.000 | (0.000) | <0.001 | 0.000 | (0.000) | 0.002 | -0.000 | (0.000) | <0.001 |
| Mon (vs. Sun) | 0.199 | (0.123) | 0.107 | 0.150 | (0.120) | 0.210 | -0.565 | (0.148) | <0.001 |
| Tue (vs. Sun) | 0.304 | (0.122) | 0.012 | 0.304 | (0.112) | 0.006 | -0.595 | (0.151) | <0.001 |
| Wed (vs. Sun) | 0.326 | (0.119) | 0.006 | 0.342 | (0.114) | 0.003 | -0.544 | (0.152) | <0.001 |
| Thu (vs. Sun) | 0.149 | (0.125) | 0.233 | 0.154 | (0.113) | 0.171 | -0.437 | (0.158) | 0.006 |
| Fri (vs. Sun) | 0.066 | (0.122) | 0.589 | 0.140 | (0.119) | 0.238 | -0.403 | (0.155) | 0.010 |
| Sat (vs. Sun) | 0.041 | (0.135) | 0.759 | -0.081 | (0.129) | 0.531 | -0.218 | (0.157) | 0.166 |
| Spring (vs. Winter) | -0.038 | (0.004) | <0.001 | -0.008 | (0.004) | 0.039 | 0.179 | (0.005) | <0.001 |
| Summer (vs. Winter) | -0.155 | (0.004) | <0.001 | -0.109 | (0.004) | <0.001 | 0.308 | (0.005) | <0.001 |
| Autumn (vs. Winter) | -0.099 | (0.004) | <0.001 | -0.054 | (0.004) | <0.001 | 0.251 | (0.005) | <0.001 |
| Year 2021 (vs. 2020) | -0.029 | (0.004) | <0.001 | -0.028 | (0.004) | <0.001 | 0.163 | (0.005) | <0.001 |
| Year 2022 (vs. 2020) | -0.096 | (0.006) | <0.001 | -0.093 | (0.006) | <0.001 | 0.324 | (0.008) | <0.001 |
| Mon*Time | -0.044 | (0.026) | 0.093 | -0.035 | (0.025) | 0.171 | 0.117 | (0.030) | <0.001 |
| Tue *Time | -0.059 | (0.026) | 0.022 | -0.062 | (0.024) | 0.009 | 0.120 | (0.032) | <0.001 |
| Wed *Time | -0.065 | (0.025) | 0.011 | -0.070 | (0.024) | 0.004 | 0.108 | (0.032) | 0.001 |
| Thu *Time | -0.025 | (0.027) | 0.353 | -0.030 | (0.024) | 0.213 | 0.086 | (0.033) | 0.009 |
| Fri *Time | -0.017 | (0.026) | 0.522 | -0.036 | (0.026) | 0.166 | 0.081 | (0.033) | 0.013 |
| Sat *Time | -0.012 | (0.029) | 0.665 | 0.012 | (0.028) | 0.667 | 0.046 | (0.033) | 0.169 |
| Mon *Time^2^ | 0.003 | (0.002) | 0.087 | 0.002 | (0.002) | 0.144 | -0.007 | (0.002) | <0.001 |
| Tue *Time^2^ | 0.004 | (0.002) | 0.032 | 0.004 | (0.002) | 0.010 | -0.007 | (0.002) | 0.001 |
| Wed *Time^2^ | 0.004 | (0.002) | 0.013 | 0.005 | (0.002) | 0.005 | -0.006 | (0.002) | 0.002 |
| Thu *Time^2^ | 0.001 | (0.002) | 0.435 | 0.002 | (0.002) | 0.226 | -0.005 | (0.002) | 0.016 |
| Fri *Time^2^ | 0.001 | (0.002) | 0.523 | 0.002 | (0.002) | 0.152 | -0.005 | (0.002) | 0.028 |
| Sat *Time^2^ | 0.001 | (0.002) | 0.588 | -0.000 | (0.002) | 0.808 | -0.003 | (0.002) | 0.204 |
| Mon *Time^3^ | -0.000 | (0.000) | 0.089 | -0.000 | (0.000) | 0.151 | 0.000 | (0.000) | 0.001 |
| Tue *Time^3^ | -0.000 | (0.000) | 0.045 | -0.000 | (0.000) | 0.013 | 0.000 | (0.000) | 0.002 |
| Wed *Time^3^ | -0.000 | (0.000) | 0.015 | -0.000 | (0.000) | 0.007 | 0.000 | (0.000) | 0.005 |
| Thu *Time^3^ | -0.000 | (0.000) | 0.503 | -0.000 | (0.000) | 0.245 | 0.000 | (0.000) | 0.027 |
| Fri *Time^3^ | -0.000 | (0.000) | 0.573 | -0.000 | (0.000) | 0.170 | 0.000 | (0.000) | 0.063 |
| Sat *Time^3^ | -0.000 | (0.000) | 0.553 | 0.000 | (0.000) | 0.896 | 0.000 | (0.000) | 0.263 |
| Constant | 0.120 | (0.102) | 0.239 | 0.156 | (0.092) | 0.090 | 0.202 | (0.122) | 0.098 |
| Random estimates |  |  |  |  |  |  |  |  |  |
| $\sigma_{1}^{2}$ | 0.000 | (0.000) | <0.001 | 0.000 | (0.000) | <0.001 | 0.000 | (0.000) | <0.001 |
| $\sigma_{0}^{2}$ | 0.666 | (0.009) | <0.001 | 0.696 | (0.009) | <0.001 | 0.645 | (0.008) | <0.001 |
| $\sigma_{01}$ | -0.005 | (0.000) | <0.001 | -0.006 | (0.000) | <0.001 | -0.005 | (0.000) | <0.001 |
| $\sigma_{\varepsilon}^{2}$ | 0.218 | (0.002) | <0.001 | 0.220 | (0.002) | <0.001 | 0.292 | (0.003) | <0.001 |
| Number of individuals (n) | 49,218 |  |  | 49,218 |  |  | 49,218 |  |  |
| Number of observations (N) | 924,367 |  |  | 922,141 |  |  | 825,410 |  |  |

*Note:* All time-invariant covariates (age, gender, ethnicity, education, area of living, living status, physical health, mental health) were included in each model, but estimates are not reported to avoid Table 2 fallacy.

**Table S4b.** Results from linear mixed-effects models testing whether associations between time and life satisfaction, worthwhile, and loneliness were moderated by day of the week.

|  | Life Satisfaction | | | Worthwhile | | | Loneliness | | |
| --- | --- | --- | --- | --- | --- | --- | --- | --- | --- |
| Fixed estimates | Coef. | SE | p | Coef. | SE | p | Coef. | SE | p |
| Time | -0.124 | (0.025) | <0.001 | -0.101 | (0.026) | <0.001 | 0.029 | (0.022) | 0.183 |
| Time^2^ | 0.008 | (0.002) | <0.001 | 0.007 | (0.002) | <0.001 | -0.002 | (0.001) | 0.201 |
| Time^3^ | -0.000 | (0.000) | <0.001 | -0.000 | (0.000) | <0.001 | 0.000 | (0.000) | 0.234 |
| Mon (vs. Sun) | -0.521 | (0.148) | <0.001 | -0.343 | (0.154) | 0.026 | 0.004 | (0.120) | 0.971 |
| Tue (vs. Sun) | -0.684 | (0.143) | <0.001 | -0.385 | (0.146) | 0.009 | 0.204 | (0.126) | 0.106 |
| Wed (vs. Sun) | -0.451 | (0.146) | 0.002 | -0.279 | (0.149) | 0.061 | 0.078 | (0.120) | 0.513 |
| Thu (vs. Sun) | -0.376 | (0.149) | 0.011 | -0.216 | (0.151) | 0.151 | -0.041 | (0.130) | 0.752 |
| Fri (vs. Sun) | -0.400 | (0.152) | 0.008 | -0.211 | (0.163) | 0.195 | -0.055 | (0.124) | 0.658 |
| Sat (vs. Sun) | -0.264 | (0.162) | 0.104 | 0.042 | (0.163) | 0.795 | 0.105 | (0.135) | 0.436 |
| Spring (vs. Winter) | 0.206 | (0.005) | <0.001 | 0.144 | (0.005) | <0.001 | -0.050 | (0.003) | <0.001 |
| Summer (vs. Winter) | 0.363 | (0.006) | <0.001 | 0.262 | (0.005) | <0.001 | -0.091 | (0.004) | <0.001 |
| Autumn (vs. Winter) | 0.318 | (0.005) | <0.001 | 0.255 | (0.005) | <0.001 | -0.072 | (0.004) | <0.001 |
| Year 2021 (vs. 2020) | 0.234 | (0.005) | <0.001 | 0.181 | (0.004) | <0.001 | -0.024 | (0.003) | <0.001 |
| Year 2022 (vs. 2020) | 0.423 | (0.008) | <0.001 | 0.331 | (0.007) | <0.001 | -0.084 | (0.006) | <0.001 |
| Mon*Time | 0.115 | (0.031) | <0.001 | 0.076 | (0.032) | 0.017 | 0.001 | (0.025) | 0.971 |
| Tue *Time | 0.143 | (0.030) | <0.001 | 0.077 | (0.030) | 0.011 | -0.037 | (0.026) | 0.155 |
| Wed *Time | 0.090 | (0.031) | 0.003 | 0.053 | (0.031) | 0.085 | -0.011 | (0.025) | 0.670 |
| Thu *Time | 0.082 | (0.032) | 0.010 | 0.045 | (0.032) | 0.156 | 0.015 | (0.027) | 0.588 |
| Fri *Time | 0.086 | (0.032) | 0.008 | 0.049 | (0.034) | 0.152 | 0.011 | (0.026) | 0.686 |
| Sat *Time | 0.062 | (0.035) | 0.074 | -0.005 | (0.034) | 0.888 | -0.024 | (0.028) | 0.399 |
| Mon *Time^2^ | -0.008 | (0.002) | <0.001 | -0.005 | (0.002) | 0.020 | -0.000 | (0.002) | 0.834 |
| Tue *Time^2^ | -0.009 | (0.002) | <0.001 | -0.005 | (0.002) | 0.018 | 0.002 | (0.002) | 0.252 |
| Wed *Time^2^ | -0.005 | (0.002) | 0.007 | -0.003 | (0.002) | 0.137 | 0.000 | (0.002) | 0.877 |
| Thu *Time^2^ | -0.005 | (0.002) | 0.012 | -0.003 | (0.002) | 0.174 | -0.001 | (0.002) | 0.433 |
| Fri *Time^2^ | -0.005 | (0.002) | 0.012 | -0.003 | (0.002) | 0.170 | -0.001 | (0.002) | 0.612 |
| Sat *Time^2^ | -0.004 | (0.002) | 0.069 | 0.000 | (0.002) | 0.886 | 0.002 | (0.002) | 0.405 |
| Mon *Time^3^ | 0.000 | (0.000) | <0.001 | 0.000 | (0.000) | 0.029 | 0.000 | (0.000) | 0.694 |
| Tue *Time^3^ | 0.000 | (0.000) | <0.001 | 0.000 | (0.000) | 0.028 | -0.000 | (0.000) | 0.380 |
| Wed *Time^3^ | 0.000 | (0.000) | 0.014 | 0.000 | (0.000) | 0.199 | 0.000 | (0.000) | 0.925 |
| Thu *Time^3^ | 0.000 | (0.000) | 0.014 | 0.000 | (0.000) | 0.196 | 0.000 | (0.000) | 0.320 |
| Fri *Time^3^ | 0.000 | (0.000) | 0.022 | 0.000 | (0.000) | 0.212 | 0.000 | (0.000) | 0.509 |
| Sat *Time^3^ | 0.000 | (0.000) | 0.075 | -0.000 | (0.000) | 0.842 | -0.000 | (0.000) | 0.442 |
| Constant | 0.119 | (0.121) | 0.328 | -0.068 | (0.127) | 0.592 | 0.077 | (0.106) | 0.470 |
| Random estimates |  |  |  |  |  |  |  |  |  |
| $\sigma_{1}^{2}$ | 0.000 | (0.000) | <0.001 | 0.000 | (0.000) | <0.001 | 0.000 | (0.000) | <0.001 |
| $\sigma_{0}^{2}$ | 0.657 | (0.008) | <0.001 | 0.662 | (0.008) | <0.001 | 0.701 | (0.006) | <0.001 |
| $\sigma_{01}$ | -0.007 | (0.000) | <0.001 | -0.007 | (0.000) | <0.001 | -0.004 | (0.000) | <0.001 |
| $\sigma_{\varepsilon}^{2}$ | 0.304 | (0.003) | <0.001 | 0.331 | (0.003) | <0.001 | 0.184 | (0.002) | <0.001 |
| Number of individuals (n) | 49,218 |  |  | 49,218 |  |  | 49,218 |  |  |
| Number of observations (N) | 929,813 |  |  | 929,810 |  |  | 921,074 |  |  |

*Note:* All time-invariant covariates (age, gender, ethnicity, education, area of living, living status, physical health, mental health) were included in each model, but estimates are not reported to avoid Table 2 fallacy.

**Figure S1.** Number of observations across time of day.


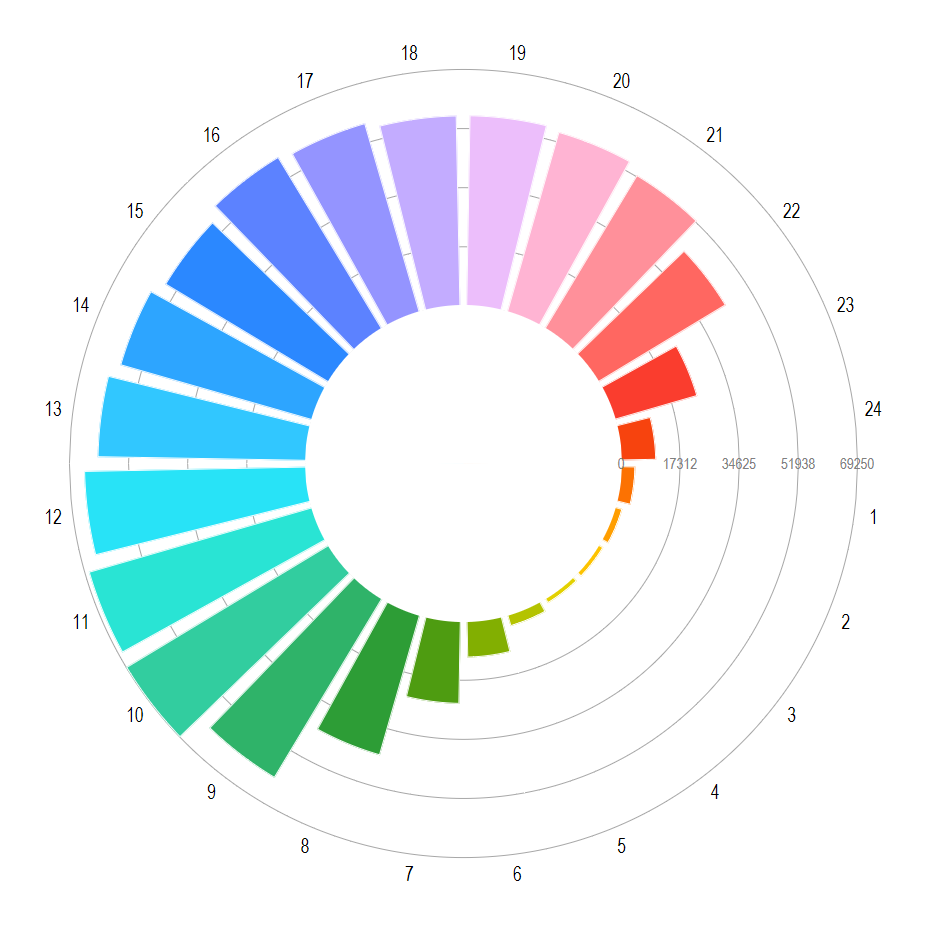

Supplement: online supplemental file 1 [file bmjment-28-1-s001.docx]
